# Increased Disease Calls for a Cost-Benefits Review of Marine Reserves

**DOI:** 10.1371/journal.pone.0051615

**Published:** 2012-12-11

**Authors:** Emma C. Wootton, Andrew P. Woolmer, Claire L. Vogan, Edward C. Pope, Kristina M. Hamilton, Andrew F. Rowley

**Affiliations:** 1 Department of Biosciences, Swansea University, Swansea, United Kingdom; 2 Salacia-Marine, Pontardawe, Swansea, United Kingdom; 3 College of Medicine, Swansea University, Swansea, United Kingdom; Norwegian Polar Institute, Norway

## Abstract

Marine reserves (or No-Take Zones) are implemented to protect species and habitats, with the aim of restoring a balanced ecosystem. Although the benefits of marine reserves are commonly monitored, there is a lack of insight into the potential detriments of such highly protected waters. High population densities attained within reserves may induce negative impacts such as unfavourable trophic cascades and disease outbreaks. Hence, we investigated the health of lobster populations in the UK’s Marine Conservation Zone (MCZ) at Lundy Island. Comparisons were made between the fished, Refuge Zone (RZ) and the un-fished, No-Take Zone (NTZ; marine reserve). We show ostensibly positive effects such as increased lobster abundance and size within the NTZ; however, we also demonstrate apparent negative effects such as increased injury and shell disease. Our findings suggest that robust cost-benefit analyses of marine reserves could improve marine reserve efficacy and subsequent management strategies.

## Introduction

The declining state of our marine environment is of global concern [1]–[2], thus there is a growing demand to increase the number of highly protected marine reserves. These reserves are No-Take Zones (NTZs) designed to protect habitats of ecological importance; with the aim of restoring ecosystem function via increased species density and biodiversity [3]–[4]. Their conservation benefit is principally documented with respect to heavily fished species, where high increases in density have been observed [5]–[7]. Such increases, however, may not always be advantageous and may hinder conservation objectives. Classical epidemiological theory predicts that high population density will increase the prevalence and intensity of pathogens [8], and both theoretical and empirical evidence support this theory [9]–[11]. High host abundance in marine reserves may, therefore, render animals vulnerable to disease [12], [13], particularly because infections can no longer be ‘fished out’ [14], [15].

The key to elucidating the impact of marine reserves is effective monitoring with implementation of robust cost-benefit analyses. However, because advocacy currently plays a prominent role in marine reserve implementation, positive data on the benefits of marine reserves, such as increased species biomass, density, size and biodiversity [4], [16], [17], drastically outweigh studies on the potential negative ‘costs’ of marine reserves, such as disease, parasitism, unfavourable trophic cascades and mass mortality [18]–[21]. Furthermore, increased population density and connectivity are pivotal drivers in the spread of disease, and as a consequence, continued advocacy may prove detrimental to the long term effectiveness of marine reserves [22], [23].

Inclusion of ‘health’ and disease status in monitoring studies may help attain equality in the cost-benefit analysis of marine reserve efficacy. In this paper, we investigate the population structure and health of the European lobster, *Homarus gammarus*, in the Marine Conservation Zone (MCZ) at Lundy Island, UK. The Lundy MCZ comprises a Refuge Zone (RZ) where pot fisheries are authorised, but trawl and net fisheries are prohibited; and a statutory marine reserve (No-Take Zone; NTZ) within which all fishing and removal of wildlife (except that required for scientific monitoring) is forbidden. During our survey, we not only recorded the gender and size of European lobsters (*Homarus gammarus*), we also documented claw loss, injury and shell disease in order to assess the stresses encountered by this species. Comparisons were made between the lobster populations in the NTZ and RZ in order to elucidate the impact of a marine reserve (NTZ) on the health of resident lobsters. *H. gammarus* is a highly profitable commercial shellfish species; hence, its sustainability is of great importance to the UK inshore fishing fleet.

Injury (such as puncture wounds and stress fractures to the exoskeleton), claw loss (resulting from autotomy) and shell disease (a bacterial infection of the exoskeleton) are sub-lethal conditions commonly exhibited by decapod crustaceans. Such health issues have been associated with deleterious impacts on behaviour, foraging, growth, reproduction and immunocompetence [24]–[26]; hence there are implications for individual survivorship as well as for population productivity, which may lessen the benefits of marine reserves.

## Materials and Methods

### Sample Site

**Figure 1:**
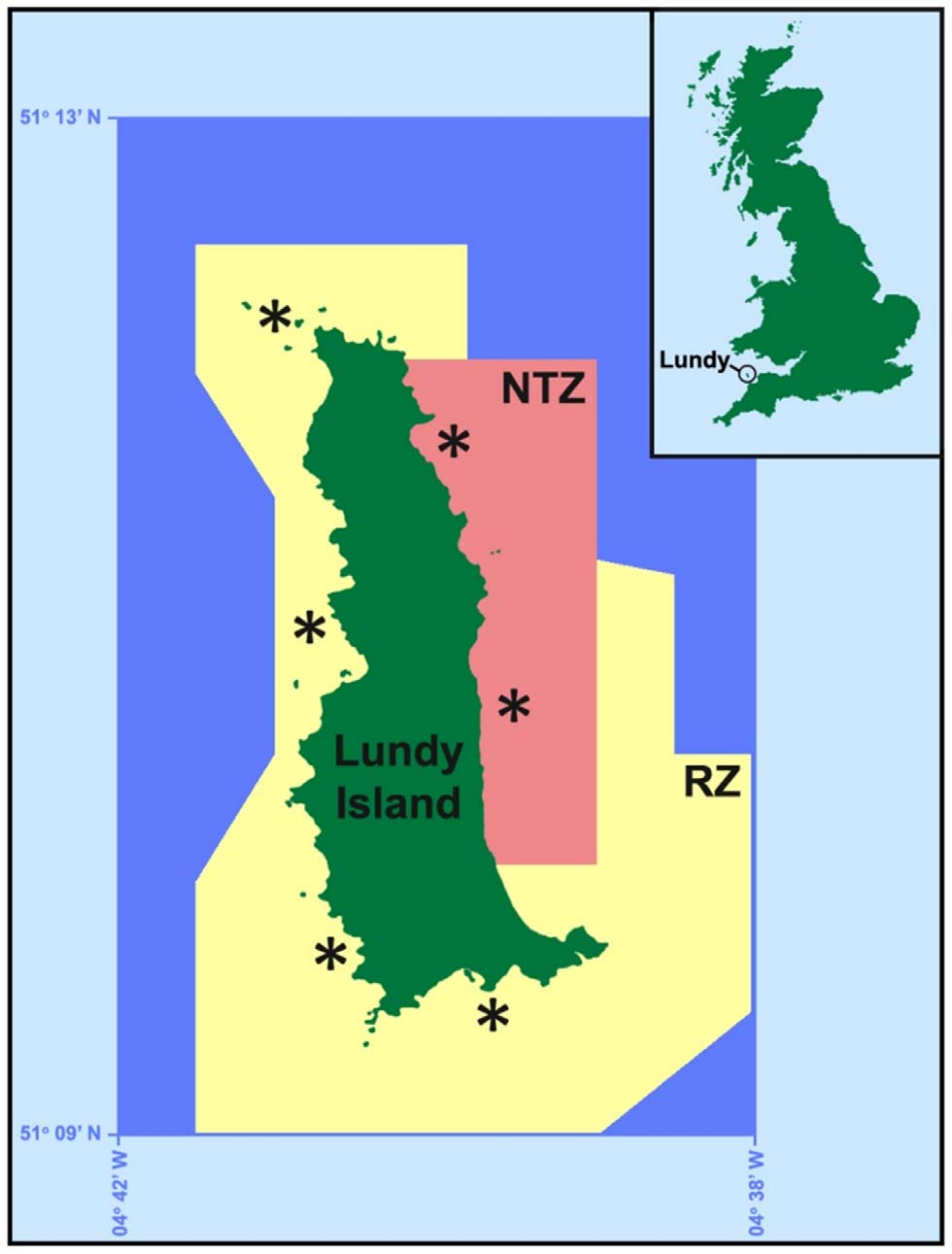
Map of Lundy Island Marine Conservation zone (MCZ), Bristol Channel, UK **(adapted from** http://www.lundymcz.org.uk/ **).** Latitude and longitude coordinates represent the MCZ boundary. RZ, Refuge Zone (yellow) where pot fisheries are authorised, but trawl and net fisheries are banned. NTZ, No-Take Zone (red) where removal of all wildlife (except that required for scientific monitoring) is prohibited; Asterisks (*) represent the sampling sites. The colour scheme (RZ = yellow; NTZ = red) is applied to all subsequent Figures.

Lundy Island (5×1.25 km) is located in the Bristol Channel, U.K. (Figure 1). Lundy and its surrounding waters (*ca*. 30 km^2^) were designated a Marine Nature Reserve (MNR) in 1986, and included a Refuge Zone (RZ; up to 1.5 km offshore; coloured yellow in Figure 1), where pot fisheries (for crabs and lobsters) were authorised, but trawl and net fisheries prohibited. However, in 2003, a statutory No-Take Zone (NTZ; 3.3 km^2^; coloured red in Figure 1), also known as a marine reserve, was imposed within the existing RZ on the Eastern shore of the Island. Within the NTZ, all fishing (including potting) and removal of wildlife (except that required for scientific monitoring) was forbidden. In January 2010, the Lundy MNR became the UK’s first MCZ under the 2009 UK Marine and Coastal Access Act. Both the RZ and NTZ are maintained within its MCZ status (http://www.lundymcz.org.uk/).

### Lobster Collection

During two sampling trips, in May (4 days) and July (4 days) 2010, European lobsters, *Homarus gammarus*, were sampled in the Lundy Island MCZ. Six sampling sites, similar to those of a previous Lundy Island survey [27], were equally spaced around the island, and were located within both the RZ and NTZ (Figure 1). At the start of each sampling trip, one string of standard baited commercial parlour pots (35 pots with escape gaps closed) was deployed at each of the six sampling sites (Figure 1). Each string was immersed (‘soaked’) for 24–48h (weather dependant), after which it was retrieved, emptied of all catch, and then re-baited and redeployed in a similar position. The process of pot deployment, soaking and retrieval was repeated throughout the duration of the sampling trip. All captured lobsters were immediately examined and returned to the water.

### Examination Procedure

Gross external parameters of each lobster (*N = *666) were recorded to assess both population structure and individual lobster health. Lobsters exhibiting exoskeletal abnormalities were digitally photographed for confirmation of their exoskeletal health status.

#### Population structure parameters

Gender (including whether females were egg-bearing) and size (carapace length, CL; mm) of each lobster were recorded to provide frequency distributions for both zones (RZ and NTZ). For size analyses, lobsters were classified as either small or large, based on the Minimum Landing Size (MLS; i.e. 90 mm CL). Small lobsters were <MLS, whilst large lobsters were >MLS. This size categorization allowed for assessment of fishing effort on population structure and lobster health.

#### Lobster health parameters

**Figure 2:**
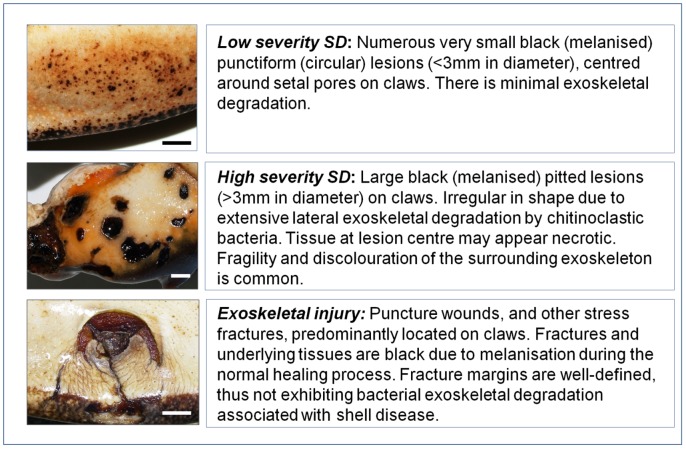
Classification of shell disease (SD) and exoskeletal injury in European lobsters, *Homarus gammarus*. These criteria were used to assess the health status of lobsters at Lundy Island in July and May 2010. Scale bar = 1 cm.

Injury, cheliped (claw) loss and shell disease were recorded in every individual. Attention was focused on the claws, cephalothorax and abdomen due to the limitations of ‘onboard’ sampling. Classification details are summarised below and in Figure 2.

Injury was classified as puncture wounds and stress fractures to the exoskeleton (Figure 2). Injuries inflicted during captivity within the lobster pot (i.e. recent, non-melanised, injury) were not recorded. Shell disease prevalence (i.e. presence/absence) was recorded in all caught lobsters, whereas shell disease severity (i.e. high/low; Figure 2) was only recorded in lobsters caught during the July survey. The absence of claws and/or the presence of dwarf claw regenerates, resulting from limb autotomy (a reflex response to predation and other threats), was recorded in all lobsters.

### Statistical Analyses

Data collected during both sampling trips (May and July) were combined, and data from sampling sites within each zone were pooled due to the small sample size from some individual sites (particularly in the RZ). Pooling of data was based on equal fishing effort (i.e. equal number of pots and ‘soak’ time), thus allowing a direct comparison between lobsters caught in NTZ and RZ. Where possible, data from each zone was further categorised according to gender and/or lobster size in order to examine the effects of gender and fishing effort on health and disease status. For frequency data analyses, Fisher’s exact tests of independence were used investigate the association between two categorised variables, in particular, the relationship between zone (RZ or NTZ) and the prevalence of an exoskeletal parameter (e.g. size, gender, injury, shell disease or claw loss etc). Such tests were also used to explore relationships between two exoskeletal parameters within one particular zone (e.g. the association between injury and shell disease within the NTZ). All tests were two-tailed and used a significance level of *α*<0.05. Comparisons of mean lobster size were carried out with unpaired T-tests (two-tailed with a significance level of *α*<0.05). All data sets were confirmed to follow a Gaussian distribution (using the Kolmogorov-Smirov test) prior to T-test analysis. Catch Per Unit of fishing Effort (CPUE) was calculated as the mean number of lobsters per pot (based on equal fishing effort, i.e. pots with identical soak times).

## Results and Discussion

### Effects of the Marine Reserve on Lobster Population Structure

**Figure 3:**
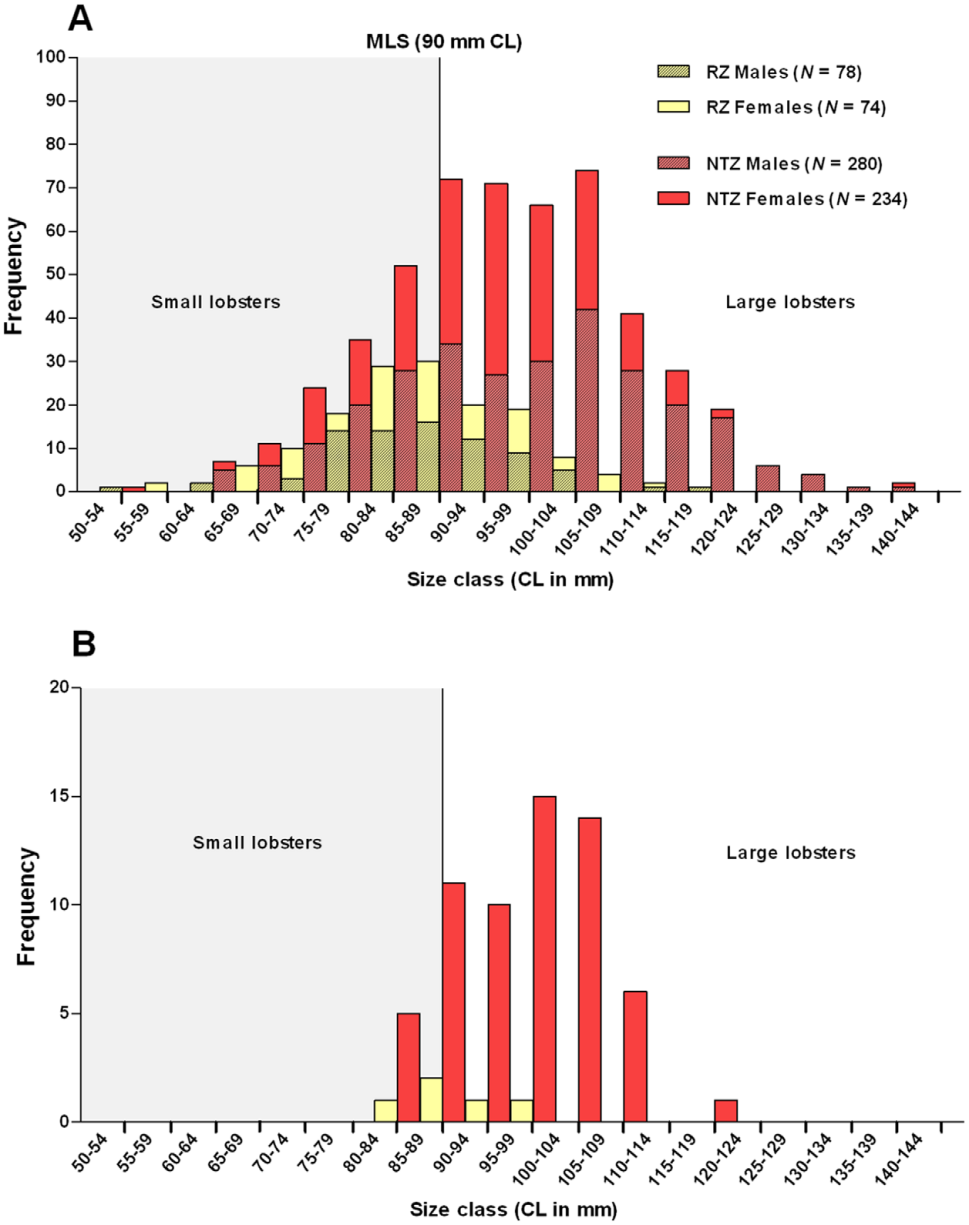
Population structures of Lundy Island lobsters. (A) Size-frequency distributions of lobsters surveyed from the Refuge Zone (RZ, coloured yellow) and the No-Take Zone (NTZ, coloured red); (B) Size-frequency distributions of ovigerous (‘berried’) female lobsters from the RZ and NTZ. MLS, Minimum Landing Size.

Overall, the catch data revealed that a greater number of lobsters were caught in the NTZ than in the RZ (NTZ = 514, RZ = 152; Figure 3a and Table S1), with the Catch Per Unit of fishing Effort (CPUE) being 7.71 times greater in the NTZ (1.364 v. 0.177; unpaired T-test, P<0.0001). A previous survey at Lundy Island in 2007 [27]; which used very similar sampling locations, revealed a 5.0-fold increase in lobster abundance in the NTZ compared with the RZ, thus the 7.71-fold increase in CPUE observed during our current study suggests the lobster population in the NTZ continued to increase between 2007–2010.

Classification of lobsters as either small (< Minimum Landing Size; MLS, i.e. <90 mm carapace length; CL) or large (> MLS, i.e. >90 mm CL) allowed for assessment of fishing effort on population structure. We found the NTZ population to comprise 74.7% large lobsters (and the RZ only 35.5%; Table S1), with a significant increase in the abundance of both large male and female lobsters in the NTZ (Fisher’s Exact test, *P*<0.0001, Figure 3a and Table S2). Most notably, the frequency of RZ lobsters rapidly declined immediately above the MLS (90 mm CL), probably as a result of substantial fishing pressure; whereas in the NTZ, lobster frequency only began to rapidly decline above 110 mm CL (Figure 3a). Although competition for pot bait within the NTZ may be skewing the data in favour of large individuals, changes in the NTZ population structure are evident. The NTZ is counteracting ‘longevity overfishing’ whereby older age classes are fished out [28]. Mean CL measurements illustrated that lobsters from the NTZ were also significantly larger than those from the RZ (unpaired T- test, 5, *P*<0.0001, Table S1) and further analysis revealed that the significant size increase was only evident in the large lobster categories (unpaired T-test, large males = *P*<0.0001; large females = *P = *0.043, Table S2).

There was no significant difference in the gender ratio (M:F) between the two zones (Table S1), however, there were significantly more egg-bearing (ovigerous) females in the NTZ than in the RZ (Fisher’s Exact test, *P*<0.0001; Figure 3b and Tables S1 and S2), and a significantly greater proportion of those lobsters were large females (Fisher’s Exact test, *P* = 0.011). Studies have shown that large female lobsters are reproductively more successful than their smaller counterparts [29], hence the greater abundance of large ovigerous females in the NTZ highlights the potential for increased egg production within the reserve. These large female lobsters may therefore help maintain numerous fisheries stocks via recruitment subsidy.

### Effects of the Marine Reserve on Lobster Health

Both injury and shell disease (SD) were observed in the NTZ and RZ, and were predominantly located on lobster claws. This is unsurprising given the active role these appendages play in foraging, burrowing and defence. Injury was consistently observed as puncture wounds and stress fractures to the claw exoskeleton; whilst SD was documented as either small punctiform lesions (low severity) or large pitted lesions in the exoskeleton (high severity; Figure 2). Severe injury and SD on the cephalothorax and abdomen were extremely rare (3/666 lobsters).

**Figure 4:**
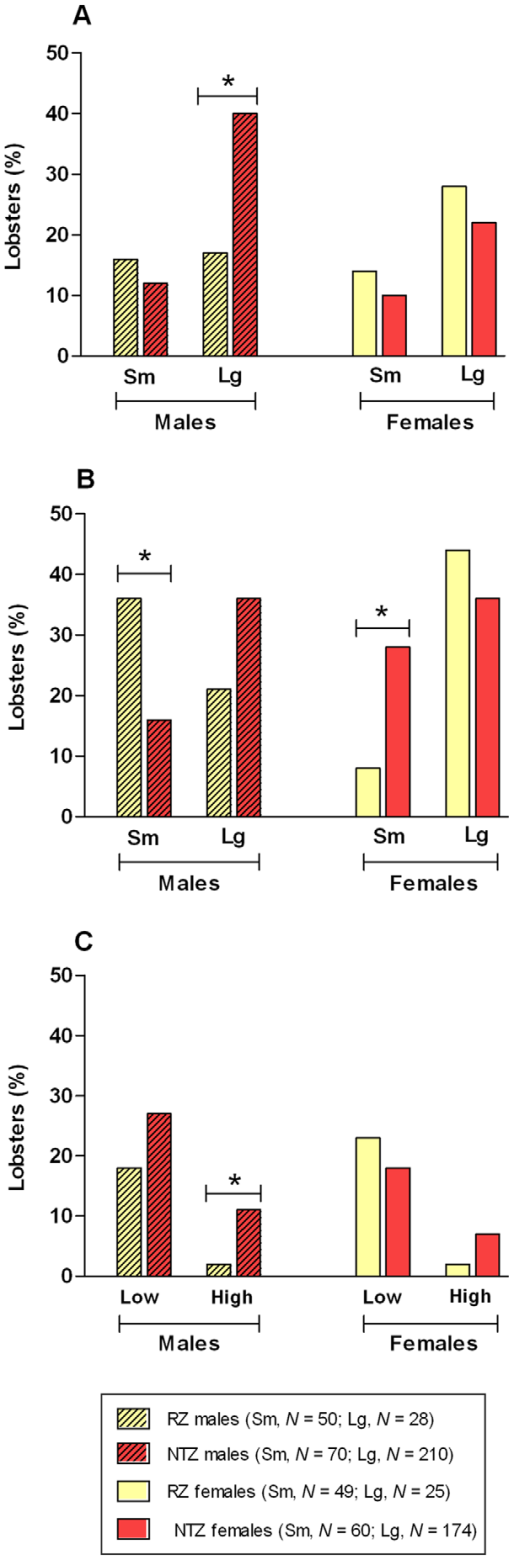
Health comparisons of Lundy Island lobsters. Prevalence of (A) Injury; (B) Shell disease; (C) Shell disease severity (low and high) in surveyed lobsters. RZ, Refuge Zone; NTZ, No-Take Zone; Sm, Small lobsters (< MLS); Lg, Large lobsters (>MLS); MLS, Minimum Landing Size. Asterisks represent significant differences (* = *P*<0.05).

An overall comparison between lobsters caught in the RZ and NTZ revealed that there was no significant difference in the prevalence of injury between the two zones (Table S1). However, further analysis of categorised lobsters (based on size and gender) demonstrated significant differences within particular groupings (Table S2). Injury was significantly more prevalent in small males from the RZ compared with small males from the NTZ (Fisher’s Exact test, *P* = 0.017; Figure 4a). This may be due to the high proportion of small male lobsters in the RZ (64.1% of RZ males); with competition for resources in this size group instigating agonistic behaviour. The lower abundance of large dominant male lobsters within the RZ probably allowed small lobsters to reside in lobster pots; hence, be captured. In contrast, injury was significantly more prevalent in small females from the NTZ compared with small females from the RZ (Fisher’s Exact test, *P* = 0.013; Figure 4a). This is of concern as small females within the NTZ are likely prerequisites for the future persistence of many sub-populations. The size and gender of injury perpetrators in each zone are unknown, however, both inter- and intra-sexual aggression have been observed in this species [30]–[31]. There were no significant differences in injury prevalence in the large size categories between the two zones (Figure 4a and Table S2).

Shell disease lesions were located on both the dorsal and ventral surface of claws, as well as on the margins (Figure 2). This is consistent with the sub-lethal ‘classical’ form of SD, rather than the lethal ‘epizootic’ form that has devastated lobster populations along the US Eastern seaboard. SD lobsters, regardless of severity, did not show clinical symptoms of disease such as lethargy and weakness. However, our passive sampling method may have neglected ailing lobsters. Overall, there was a significantly higher prevalence of SD in the NTZ (Table S1), and analysis of categorised data provided further insight (Table S2). For example, SD was significantly more prevalent in large males from the NTZ compared with large males from the RZ (Fisher’s Exact test, *P* = 0.036; Figure 4b and Table S2). Large male lobsters are dominant within lobster population hierarchies [32], so will be defending their resources from subordinates, particularly in high density environments. Claws are the lobsters’ principle weapon for defence, so will sustain much damage. Exoskeletal damage, especially to the waxy outer epicuticle, will allow entry to potential causative agents of shell disease, such as chitinoclastic bacteria and fungi, to the underlying procuticle, thus inducing exoskeletal infection and lesions [33]. High lobster abundance in the NTZ suggests that SD pathogens will be more prevalent in the local environment; and the common association of lobster SD with high host density [34]–[35] supports this theory. Furthermore, decreasing moult frequency in large lobsters results in longer retention of the exoskeleton, which in turn increases the potential for SD instigation and/or progression. There was no significant difference in SD prevalence between small males of the NTZ and RZ (Figure 4b; Table S2). Female lobsters (both small and large) also did not exhibit significant differences in SD prevalence between the two zones (Figure 4b and Table S2).

Our investigations into the severity of SD revealed that, overall, low severity SD was the most common form of the disease, with no significant difference between the two zones (Table S3). There was also no significant difference in low severity SD prevalence in categorised male and female lobsters (Figure 4c and Table S4). Little information exists on SD in European lobsters; hence, the majority of our knowledge is obtained from the American lobster, *Homarus americanus*. At Lundy Island, low severity SD was synonymous to Grade I Impoundment SD [35] and early-stage burnt (or rust) spot SD in *H. americanus* [34]. It does not appear detrimental to Lundy lobsters and would probably be lost during the next moult. In contrast, high severity SD was less prevalent than low severity SD in lobsters surveyed during our Lundy Island study (Table S3), but significantly more lobsters in the NTZ exhibited this form of the disease (Fisher’s Exact test, *P* = 0.006). Notably, ‘fishing out’ of large lobsters from the RZ appears to be suppressing SD disease severity in this zone, with only 2 individuals (one male and one female) from this zone exhibiting high severity SD (Table S4). Analysis of separate genders revealed a significantly higher prevalence of high severity SD in male lobsters from the NTZ compared with male lobsters from the RZ (Fisher’s Exact test, *P* = 0.030; Figure 4c). There was, however, no significant difference in prevalence of high severity SD in female lobsters (Figure 4c and Table S4).

There is uncertainty in determining the aetiology of high severity SD in Lundy lobsters. The large pitted lesions may have originated from low severity shell disease, with small punctiform lesions progressively spreading and merging to form large continuous necrotic lesions [35]. We observed simultaneous expression of low and high severity SD in lobsters and there was a significant association between presence of low and high severity SD in lobsters from the NTZ (Fisher’s Exact test, *P*<0.0001). Lobsters exhibiting low severity SD were 4.08 times more likely to simultaneously exhibit high severity SD (relative risk calculated from proportional ratios). Alternatively, the lesions may have derived from infected injuries. We discovered puncture wounds exhibiting SD, suggesting that fighting injuries and SD are interrelated. This theory is supported by the significantly higher prevalence of high severity SD in male lobsters from the NTZ (Figure 4c) and a statistically significant association between high severity SD and injury in these animals (Fisher’s Exact test, *P* = 0.010). Injured NTZ males were 3.15 times more likely to possess high severity SD than uninjured NTZ males (relative risk calculated from proportional ratios). Further research, however, is required to fully understand the symptoms, aetiology and epidemiology of SD at Lundy Island.

Loss of claws (through autotomy) occurs in response to predation, injury, problematic moulting, unfavourable environmental conditions and fishing activities [24]. Although, there are immediate survival benefits, there are long-term functional costs that can potentially impact at the population level. During our survey, we observed claw loss (93.2% was single claw loss) in both the RZ and NTZ, and its prevalence did not significantly differ between the two zones (Tables S1 and S2). This suggests that the NTZ is not counteracting the claw loss induced by stressors in the RZ.

### Implications for Population Persistence and Connectivity

The present study provides a ‘snap shot’ of the lobster populations at Lundy Island. The possibility that our passive ‘pot’ sampling method may have introduced sample bias [36] is taken into consideration when interpreting our results. The significantly greater CPUE, lobster size and abundance of ovigerous females observed within the Lundy NTZ are classic beneficial outcomes of marine reserve implementation on commercial species, in particular, lobsters [7], [27], [37]. However, although lobster abundance has increased within the Lundy NTZ; over time, the lobster density is likely to stabilise due to resource limitation. Consequently, there is potential for ‘spillover’ into surrounding waters through density-dependant migration [38]. Fisheries benefits of adult ‘spillover’ from marine reserves into adjacent fished areas are controversial and currently very difficult to empirically prove [22], [23], [39]. Some studies have implied adult lobster ‘spillover’ from marine reserves [40], [41] including at Lundy Island [27], however, the studies underestimated the complexities involved in proving such a hypothesis. ‘Spillover’, or export, of larvae from marine reserves is often considered more beneficial because of the potential for wide dispersal and its role in metapopulation persistence and connectivity [39], [42]. This is particularly pertinent in European lobsters, where adults have a home range of just a few kilometres but larvae can reside in the plankton for several weeks before settling into distant sub-populations. Although there were significantly more large ovigerous females in the Lundy NTZ, there was also a significantly higher prevalence of injury in small female lobsters. Both these observations warrant further monitoring if export of lobster larvae from this reserve is to be maximised.

We also revealed a significant increase in the prevalence and severity of SD in the NTZ. Severely infected individuals did not show clinical signs of disease suggesting that, at present, SD is not highly detrimental to Lundy lobster populations. However, alternative sampling techniques should be investigated in order to confirm the absence of ailing lobsters; which if present, would not be entering lobster pots and thus be sampled. In the NTZ, high severity SD was significantly associated with injury, with injured male lobsters 3.15 times more likely to possess the high severity form of SD. This may be of concern for marine reserves within which high lobster densities may stimulate agonistic behaviour of this highly territorial species. Lobsters prefer to establish rank with local residents rather than migrate to a new location with unfamiliar conspecifics. This is known as the residency-sociality hypothesis [43].

Injury and SD can also induce secondary effects on lobsters, with repercussions on foraging, feeding, moulting and territorial defence [24]–[26]. There is also potential for pathogens to enter through sites of injury and SD, and establish internal infections. This is of particular concern for infectious disease, which under high lobster densities and related stresses can induce considerable mortality [26]. Additional health monitoring studies, both in the Lundy NTZ and other marine reserves, would help establish whether our findings on lobster health at Lundy Island are directly related to the reserve itself or, in fact, a result of other confounding factors. This is pertinent given an observed reduction in tail fan necrosis in spiny lobsters within a New Zealand marine reserve [44], thus revealing a positive impact of a marine reserve on crustacean health.

### Conclusions and Wider Implications

Our study highlights the cost-benefits of a temperate marine reserve. The observed costs, such as increased injury and shell disease in lobsters, currently appear to be inflicting minimal adverse effects on their host, whilst the observed benefits, such as increased size and abundance, may be exerting positive impacts on both fisheries and conservation. The cost-benefits of such highly protected areas, however, are subjective, depending upon the objective being assessed. For example, within the Lundy NTZ, the significant increase in lobsters has been accompanied by a significant decrease in the abundance of other crustacean species ([27]; Wootton et al, unpublished data). This has been considered to result from predation and competition by lobsters [27]; with this crustacean now fulfilling the role of top predator since the demise of large predatory demersal fish, such as cod, and the prohibition of human fishing activity in the NTZ. Evidence of such trophic cascades in marine reserves, and the corresponding potential for reduction in species density and diversity [45]–[46], emphasises that benefits of marine reserves may not be equally distributed among species [21]. Robust cost-benefit analyses may therefore help solve some of the ambiguities surrounding the consequences of marine reserve implementation.

The on-going debate on marine reserve efficacy is challenging. Finding a compromise between species conservation, food security, and the social, economic and cultural requirements of coastal communities is complex. In some instances, insufficiently-evidenced policies on marine reserve implementation, based on advocacy as much as upon science, may be underestimating the ecological and socio-economic impacts of conservation science [23], [47]. A valuable alternative to highly protected marine reserves is ecosystem-based management [48]–[51], whereby marine spatial planning (which collectively assesses all users of marine resources) is combined with ecosystem resource management (which co-manages an ecosystem as a whole, and integrates all social, economic and environmental demands) This strategy is thought to deliver greater biodiversity and fisheries gains, whilst avoiding the risk of unforeseen detrimental consequences and conflict, thus securing the future productivity of our marine environment.

## Supporting Information

Table S1 **Overall population and health differences between the lobster populations of the Refuge Zone (RZ) and No-Take Zone (NTZ) at Lundy Island, UK.** Lobsters were sampled in May and July 2010. Significant differences between the RZ and NTZ are highlighted in blue.
(PDF)

Table S2 **Data on population statistics and health of lobsters surveyed at Lundy Island, UK in May and July 2010.** Lobsters are categorised according to gender, size and zone in order to assess the impact of a marine reserve (i.e. NTZ) on the population structure and health of resident lobsters. Significant differences are highlighted in blue.
(PDF)

Table S3 **Overall comparison of shell disease severity in Lundy Island lobsters (Refuge zone vs. No-take Zone) surveyed during July 2010.** Significant differences are highlighted in blue.
(PDF)

Table S4 **Data on shell disease severity in lobsters surveyed at Lundy Island during July 2010.** Lobsters are categorised according to zone and gender in order to assess the impact of a marine reserve (i.e. No-Take Zone) on the severity of shell disease in resident lobsters. Significant differences are highlighted in blue.
(PDF)
