# Correction: Increased Disease Calls for a Cost-Benefits Review of Marine Reserves

**DOI:** 10.1371/annotation/437c179f-51b6-414a-b5e2-719b6011fc50

**Published:** 2013-06-04

**Authors:** Emma C. Wootton, Andrew P. Woolmer, Claire L. Vogan, Edward C. Pope, Kristina M. Hamilton, Andrew F. Rowley

The island outline in Figure 1 is inaccurate. The following link contains the corrected Figure 1 file: 

**Figure:**
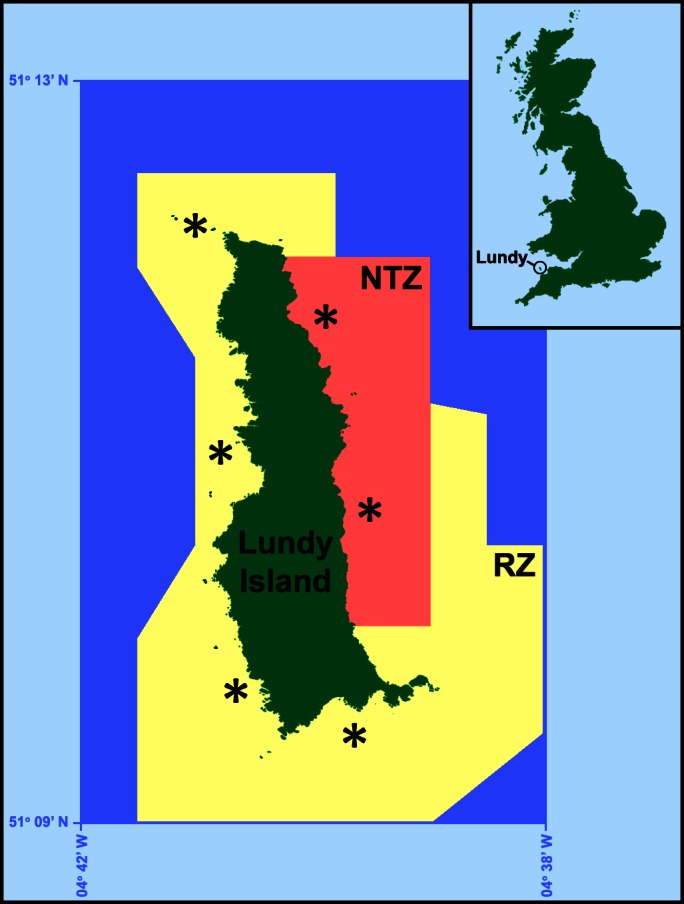


.

