# Supplementary material for: Increased Disease Calls for a Cost-Benefits Review of Marine Reserves
Source: PLoS One. 2012 Dec 11;7(12):e51615. doi: 10.1371/journal.pone.0051615 (PMC3519872; doi:10.1371/journal.pone.0051615)
Supplement: Table S1 — Overall population and health differences between the lobster populations of the Refuge Zone (RZ) and No-Take Zone (NTZ) at Lundy Island, UK. Lobsters were sampled in May and July 2010. Significant differences between the RZ and NTZ are highlighted in blue. (PDF) [file pone.0051615.s001.pdf]

Table S1. Overall population and health differences between the lobster populations of the Refuge Zone (RZ) and No-Take Zone (NTZ) at Lundy Island, UK. Lobsters were sampled in May and July 2010. Significant differences between the RZ and NTZ are highlighted in blue.

Zone	Population parameter				Health parameter (prevalence)		
	Large (>MLS) lobsters (%)	Mean size (CL in mm + SE)	Gender ratio (M : F)	Ovigerous females (% females)	Injury (%)	Shell disease (%)	Claw loss (%)
**RZ** *N* = 152	**35.5**	**86.24 + 0.91**	**1 : 0.95**	**6.8**	**25.5**	**17.8**	**9.9**
	$P < 0.0001$ ***	$P < 0.0001$ ***	$P = 0.518$	$P < 0.0001$ ***	$P = 0.134$	$P = 0.023$ *	$P = 0.768$
**NTZ** *N* = 514	**74.7**	**98.64 + 0.59**	**1 : 0.84**	**30.8**	**32.5**	**26.8**	**11.3**

RZ; Refuge Zone; NTZ, No-Take Zone; MLS, Minimum Landing Size; CL, Carapace length; SE; Standard error; M, Male; F, Female; %, percentage of lobsters from corresponding zone; *, $P < 0.05$; ***; $P < 0.001$.
