# Supplementary material for: Increased Disease Calls for a Cost-Benefits Review of Marine Reserves
Source: PLoS One. 2012 Dec 11;7(12):e51615. doi: 10.1371/journal.pone.0051615 (PMC3519872; doi:10.1371/journal.pone.0051615)
Supplement: Table S2 — Data on population statistics and health of lobsters surveyed at Lundy Island, UK in May and July 2010. Lobsters are categorised according to gender, size and zone in order to assess the impact of a marine reserve (i.e. NTZ) on the population structure and health of resident lobsters. Significant differences are highlighted in blue. (PDF) [file pone.0051615.s002.pdf]

Table S2. Data on population statistics and health of lobsters surveyed at Lundy Island, UK in May and July 2010. Lobsters are categorised according to gender, size and zone in order to assess the impact of a marine reserve (i.e. NTZ) on the population structure and health of resident lobsters. Significant differences are highlighted in blue.

Lobster category	Zone	Population Parameter: Frequency (%)		Population Parameter: Mean size (CL in mm ± SE)		Population Parameter: Ovigerous females (% females)		Health parameter (prevalence): Injury (%)		Health parameter (prevalence): Shell Disease (%)		Health parameter (prevalence): Claw loss (%)	
**Small males (<MLS)**	RZ (*N*=50)	**32.9**	*P* < 0.0001 ***	**80.76 ± 1.04**	*P* = 0.370	**NA**	NA	**36.0**	*P* = 0.017 *	**16.0**	*P* = 0.791	**9.0**	*P* = 1.00
	NTZ (*N*=70)	**13.6**		**81.89 ± 0.76**		**NA**		**15.7**		**12.9**		**11.4**	
**Large males (>MLS)**	RZ (*N*=28)	**18.4**	*P* < 0.0001 ***	**97.17 ± 1.12**	*P* < 0.0001 ***	**NA**	NA	**21.4**	*P* = 0.142	**17.9**	*P* = 0.036 *	**17.9**	*P* = 0.176
	NTZ (*N*=210)	**40.9**		**106.80 ± 0.74**		**NA**		**36.2**		**39.0**		**9.0**	
**Small females (<MLS)**	RZ (*N*=49)	**32.2**	*P* < 0.0001 ***	**79.14 ± 1.23**	*P* = 0.073	**6.1**	*P* = 0.728	**8.2**	*P* = 0.013 *	**12.2**	*P* = 0.765	**8.2**	*P* = 0.542
	NTZ (*N*=60)	**11.7**		**81.73 ± 0.82**		**8.3**		**28.3**		**10.0**		**13.3**	
**Large females (>MLS)**	RZ (*N*=25)	**16.4**	*P* < 0.0001 ***	**98.12 ± 1.14**	*P* = 0.043 *	**8.0**	*P* = 0.017 *	**44.0**	*P* = 0.509	**32.0**	*P* = 0.456	**4.0**	*P* = 0.322
	NTZ (*N*=174)	**33.8**		**101.40 ± 0.60**		**32.2**		**36.2**		**23.6**		**13.2**	

RZ; Refuge zone, NTZ; No-Take zone; MLS, Minimum landing size (90 mm CL); CL; carapace length; %, percentage of lobsters from corresponding zone, NA, not applicable;
*****, *P* < 0.05; ***, *P* < 0.0001
