# Supplementary material for: Increased Disease Calls for a Cost-Benefits Review of Marine Reserves
Source: PLoS One. 2012 Dec 11;7(12):e51615. doi: 10.1371/journal.pone.0051615 (PMC3519872; doi:10.1371/journal.pone.0051615)
Supplement: Table S3 — Overall comparison of shell disease severity in Lundy Island lobsters (Refuge zone vs. No-take Zone) surveyed during July 2010. Significant differences are highlighted in blue. (PDF) [file pone.0051615.s003.pdf]

Table S3. Overall comparison of shell disease severity in Lundy Island lobsters (Refuge zone vs. No-take Zone) surveyed during July 2010. Significant differences are highlighted in blue.

Zone	Shell disease severity			
	Low (%)		High (%)	
RZ (*N* = 121)	20.7	P = 0.612	1.7	P = 0.006 **
NTZ (*N* = 324)	23.1		9.0	

RZ; Refuge Zone; NTZ, No-Take Zone; %, percent age of lobsters from corresponding zone; **, P < 0.01.
