# Supplementary material for: Increased Disease Calls for a Cost-Benefits Review of Marine Reserves
Source: PLoS One. 2012 Dec 11;7(12):e51615. doi: 10.1371/journal.pone.0051615 (PMC3519872; doi:10.1371/journal.pone.0051615)
Supplement: Table S4 — Data on shell disease severity in lobsters surveyed at Lundy Island during July 2010. Lobsters are categorised according to zone and gender in order to assess the impact of a marine reserve (i.e. No-Take Zone) on the severity of shell disease in resident lobsters. Significant differences are highlighted in blue. (PDF) [file pone.0051615.s004.pdf]

Table S4. Data on shell disease severity in lobsters surveyed at Lundy Island during July 2010. Lobsters are categorised according to zone and gender in order to assess the impact of a marine reserve (i.e. No-Take Zone) on the severity of shell disease in resident lobsters. Significant differences are highlighted in blue.

**Lobster category**	**Zone**	**Shell disease severity (prevalence)**			
		Low (%)		High (%)	
Males	RZ (N = 60)	**18.3**	P = 0.226	**1.7**	P = 0.030 *
	NTZ (N =176)	**27.3**		**10.8**	
Females	RZ (N = 61)	**23.0**	P = 0.448	**1.6**	P = 0.182
	NTZ (N = 148)	**18.2**		**6.8**	

RZ, Refuge zone; NTZ, No-take zone; %, percentage of lobsters from corresponding zone ; * ; $P < 0.05$
